# Evaluation of the Shape Memory Effect of Random and Aligned Electrospun Polyurethane Mats with Different Fibers Diameter

**DOI:** 10.3390/polym14245468

**Published:** 2022-12-13

**Authors:** Ewa Kijeńska-Gawrońska, Adrian Maliszewski, Monika Bil

**Affiliations:** 1 Centre for Advanced Materials and Technologies CEZAMAT, Warsaw University of Technology, 02-822 Warsaw, Poland; 2 Faculty of Materials Science and Engineering, Materials Design Division, Warsaw University of Technology, 02-507 Warsaw, Poland

**Keywords:** shape memory, smart fibers, electrospinning, polyurethanes

## Abstract

Fibrous shape memory scaffolds composed of thermoplastic polyurethane based on a mixture of polycaprolactone diols were fabricated. The effect of the fiber diameter and arrangement– random (rPU) or aligned (aPU), on crystallinity, mechanical properties, and shape memory was analyzed. The diameters of the fibers were controlled by changing the concentration of polyurethane (PU) solutions in the range of 5% to 16% and fibers alignment by utilization of different collectors. The chemical structure was confirmed by Fourier Transformed Infrared spectroscopy (FTIR), crystallinity was evaluated based on differential scanning calorimetry (DSC,) and mechanical properties were measured by the tensile test. Additionally, shape memory programming was performed using a dynamic mechanical analyzer (DMA), and shape recovery was evaluated in the air and in the water environment. DSC results showed that the electrospinning process did not change the crystallinity or melting temperature of synthesized thermoplastic polyurethanes. The melting temperature of the crystalline switching segments was around 26–27 °C, and the crystalline phase of hard segments was around 130 °C. Shape memory properties were analyzed in the contest of the fiber diameter and alignment of the fibers, while changes in the fibers’ diameters from 360 nm to 1760 nm did not result in significant changes in shape recovery coefficient (Rr) especially evaluated in the air. The longitudinal fiber alignment enhanced mechanical and shape recovery to up to 96% for aPU, with the highest fiber diameter when evaluated in water.

## 1. Introduction

Shape-memory polymers (SMP) are a group of polymers possessing the ability to change their shape between temporary and permanent in a definite manner [1]. The largest group of SMP are polymers with thermal shape memory. The adaptive properties of these polymers are displayed when exposed to temperature changes [2].

The temporary shape of SMP is usually achieved by applying sufficient mechanical force to deform the permanent shape during the programming process [3]. The programming process must be carried out using the appropriate conditions—heating above the transition temperature (T_trans_), which is correlated with the glass transition (T_g_) or melting temperature T_m_ of switching segments and fixing through crystallization or vitrification by lowering the temperature below T_trans_ [4]. When the applied stress is released, the polymeric structure maintains its temporary shape until a sufficient temperature is applied, which results in the recovery of the permanent shape.

Among the class of polymeric materials that exhibit thermal shape memory effect, a large group is polyurethanes (PU). The PUs with shape memory properties is of interest in versatile sectors of industry, such as medicine [5], actuators and sensors [6,7], and textiles [8], due to their specific segmental macromolecular structure that may be modified in a broad range [9,10,11] and thus provide final polymers with various properties. Due to the lack of mixing of the polar urethane rigid segments with the flexible polyester, polyether or polycarbonate segments, which are less polar, a binary system consisting of hard and soft domains is formed within the polyurethane network. Both types of domains possess different characteristic temperatures, and in such structures, the rigid domains are responsible for retaining the original state, while the soft domains provide the ability to fix the temporary shape [12].

Moreover, the possibility of utilization of various substrates for PUs synthesis enables transition temperature’s tailoring to the requirements of the specific applications [13,14]. Additionally, the other structural characteristics recognized as critical for the shape memory effect (SME), such as crystallinity, the number of hard domains, and the extent of the specific physical interactions, such as hydrogen bonding, may be adjusted during synthesis [15].

The formation of the nonwovens with aligned fibers within their structure has an additional and significant effect on the mechanical properties and effectiveness of the shape memory of the polyurethanes [9,16]. The changes in the macroscopic properties are correlated with mats’ morphology, such as fiber diameter [17,18], but also with some changes in macromolecule orientation that may be different for bulk and nano-diameter fibers. For example, some authors [19,20,21] reported that electrospinning parameters influenced the crystallization process of PLA, PCL, and polyurethane-based on PLA-PCL copolymers, which is essential for the shape memory effect.

One of the popular methods allowing for obtaining aligned fibers from polymeric solutions or melts is electrospinning [22]. Electrospinning from polymeric, composite, or blend solutions uses an electrostatic field to induce Coulomb forces within the solution. Due to repulsive forces between solution molecules, stretched polymer jets are formed, which move in accordance with the forces of an electrostatic field, resulting in a fibrous structure formed on the collector [23]. Nevertheless, the process requires optimization in terms of utilized solvents, the concentration of the polymer, high voltage, solution feed rate, etc., to obtain structures with desired dimensions. In order to obtain an aligned structure of the electrospun mat, it is necessary to collect the fibers on a collector that ensures the alignment of the fibers in one direction. For this rotating drum, the collector can be used, which, when rotating with sufficiently high speed, allows for the fabrication of mats with highly aligned structures [24,25,26].

In the presented study, we focused on the optimization of the electrospinning of thermoplastic polyurethane in order to obtain highly aligned and random structures and to evaluate the impact of the fibers’ diameters and their arrangement within the structure on the shape memory properties of the produced mats. To the best of the authors’ knowledge, this is the first time where simultaneously both fibers’ features are evaluated in terms of their impact on the shape memory effect in polyurethanes.

## 2. Materials and Methods

### 2.1. Materials

**Figure 1:**
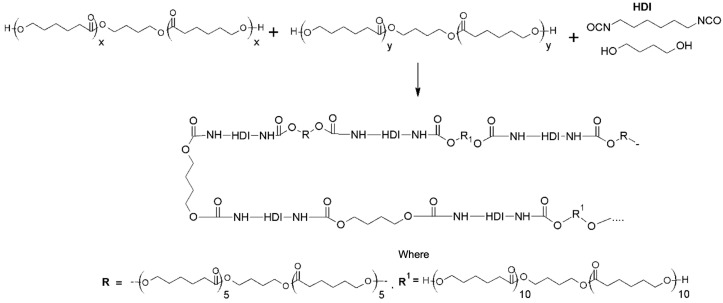
Reaction scheme.

Polyurethane (PU) utilized for fibers’ fabrication in this study has been synthesized using two PCL oligomers with different molecular weights—R, R^1^, and hexamethylene diisocyanate (HDI) according to the synthesis scheme presented in Figure 1. Polyols, after dehydration, were mixed with diisocyanate and chain extender under a nitrogen atmosphere at 60 °C. Next, synthesis was performed at 80 °C for 8 h after that polymer solution was precipitated in methanol and dried under a vacuum at 60 °C for 24 h.

1,1,1,3,3,3,-hexafluoro-2-propnaol has been purchased from Fluorochem (Glossop, UK). All reagents utilized for the synthesis of the PU were purchased from Sigma Aldrich (Burlington, MA, USA) and Chempur (Piekary Śląskie, Poland).

### 2.2. Electrospinning of Polyurethane Mats with Random and Aligned Structure

Mats with a random arrangement of the fibers within the structures have been fabricated using a nonmoving collector, which allows receiving jet to form according to the action of the resulting electrostatic field. The process was carried out at a distance of 15 cm between the needle and the collector and at a solution feed rate of 1 mL/h. The spinning voltage was adjusted to the respective solution concentrations and was 11 kV for 5% and 13 kV for 12% and 16%, respectively. The obtained nanofibers were subjected to drying for 24 h in a vacuum dryer at 50 mbar and at 25 °C.

The alignment of the nonwovens was achieved by using a cylindrical collector moving at 3000 rpm. The electrospinning process was carried out at a distance of 13 cm with a feed rate of 1 mL/h. The voltages were 21 vkV for 12% and 16% and 20 kV for 5%, respectively. The obtained nanofibers were subjected to drying for 24 h in a vacuum dryer at 50 mbar and at a temperature of 25 °C.

A volume of 1.0 mL of the solution was used for each electrospinning.

NANON-01 apparatus (MECC, Fukuoka, Japan) was used for conducting electrospinning.

### 2.3. Characterization of the Electrospun Mats

#### 2.3.1. Morphology Evaluation

Analysis of the average fiber diameter for individual fibers and determination of the degree of the alignment of the fibers in aligned structures was carried out on a Phenom ProX scanning electron microscope (SEM, PhenomX, Eindhoven, The Netherlands) after sputter coating with 7 nm layer of gold. The images were taken in backscattered electron (BSE) mode at 10 kV. Analysis of the obtained fibers was carried out using ImageJ software. Evaluation of the degree of the fibers’ alignment was performed using the Directionality module. Five images, each with a magnification of 5000× after the binarization process, were used for the alignment analysis. Evaluation of the fiber alignment was performed using Fourier spectral decomposition.

#### 2.3.2. Molecular Structure

The chemical structure of PU and nanofibrous mats was carried out using Fourier transform infrared spectrophotometer (Thermo Fisher Scientific model Nicolet 6700, Waltham, MA, USA) in the attenuated total reflectance (ATR) mode. Each sample was scanned 64 times at a resolution of 4 cm^−1^ over the frequency range of 4000–400 cm^−1^.

#### 2.3.3. Thermal Analysis

Thermal analysis was performed using a TA Q2000 differential scanning calorimeter of (DSC, TA Instruments, New Castle, DE, USA). The analysis allowed us to determine the temperatures characteristic of the tested materials and to confirm the lack of influence of the used solvent and the method of manufacturing the nonwovens on their transformation temperatures. DSC analysis was performed at the heat-cool-heat test with a heating rate of 10.00 °C/min and a cooling rate of 5.00 °C/min. Analysis was performed under a nitrogen atmosphere and within a temperature range of −80 °C −200 °C. Characteristic glass transition temperature (Tg), crystalline phase melting temperature for soft PCL segments (Tm_s_) and hard segments (Tm_h_), and the enthalpy of fusion ΔH J/g were evaluated from the second cycle heating curve. The degree of crystallinity was calculated as ΔHm/ΔH^0^m, where ΔHm is the enthalpy of melting of PCL and ΔH^0^m is the enthalpy of melting of fully crystalline PCL, which is 136 J/g [27].

#### 2.3.4. Surface Hydrophilicity

The hydrophilic properties of the fabricated mats were tested on an OCA 20 goniometer (DataPhysics, Filderstadt, Germany) using the sessile drop method. In order to ensure adequate statistics, nine measurements were used for each sample. Analysis of the surface allowed the determination of the water contact angle, directly translating into the material’s adhesion properties.

#### 2.3.5. Mechanical Properties Evaluation

The tensile stress test of the mats was carried out on a tabletop tensile machine INSTRON 5943 (size of tested samples 5 mm × 25 mm). Five specimens of each type were tested to determine the average strength and elongation at break. The specimens were subjected to tension at a rate of 5 mm/min. The results obtained from the tensile test were used to characterize the strength of the material and to determine the effect of the alignment of the fibers within the structure on their tensile strength. In the case of the aligned fibers, the strain was applied along and parallel to the fiber’s alignment.

#### 2.3.6. Shape Memory Effect Analysis

Characterization of the shape memory effect of the obtained fibrous mats was carried out on a DMA Q800 (TA Instruments) dynamic mechanical analyzer, and the last third cycle recovery step was performed in water. First, samples in the shape of strips were mounted in tensile clamps of the DMA analyzer and pre-heated to 40 °C, and then a strain deformation of 100% was applied (*ε_m_*). The strain was then held constant while the sample was cooled down to −2 °C at a rate of 5 °C/min and kept at this temperature for 15 min. The strain was then released (*ε_u_*), and the sample was heated up to 40 °C while the length recovery was monitored (*ε_p_*). In the last third cycle, the sample after the cooling step was released from the clamps and immersed in water at 40 °C. After 15 min, it was removed from the water, and the final length after recovery was measured.

Shape recovery ratio (*R_r_*) and shape fixing ratio (*R_f_*) were calculated using equations:

$$R_{r\;}=\frac{\varepsilon_{m}\;\left(N\right)-\varepsilon_{p}\left(N\right)}{\varepsilon_{m}\;\left(N\right)-\varepsilon_{p\;}\left(N-1\right)}\times 100,\;R_{f\;}=\frac{\varepsilon_{u}\;\left(N\right)}{\varepsilon_{m\;\left(N\right)}}\times 100$$

where: *ε_m_* (*N*)—maximum strain, *ε_p_*—strain after recovery, *ε_u_* (*N*) is the sample’s strain immediately following unloading.

## 3. Results and Discussion

Performed research was carried out in three stages. The first involved optimizing the electrospinning process of the nonwovens with an aligned and random arrangement of the fibers from synthesized with shape memory properties. This stage was completed after obtaining mats with two types of structures, random from 5% (rPU5), 12% (rPU12), and 16% (rPU16) concentrated solutions, respectively, and aligning orientated fibers from 5% (aPU5), 12% (aPU12) and 16% (aPU16) concentrated solutions, respectively. In the next step, characterization of the material and fabricated structures have been conducted. This stage included thermal and structural analysis, examination of the wetting angle and the diameters, and alignment of the fibers forming the mats. In the third stage, mechanical tests and shape memory effect analysis were conducted.

### Chemo-Physical Properties of the Obtained Electrospun Mats

**Figure 2:**
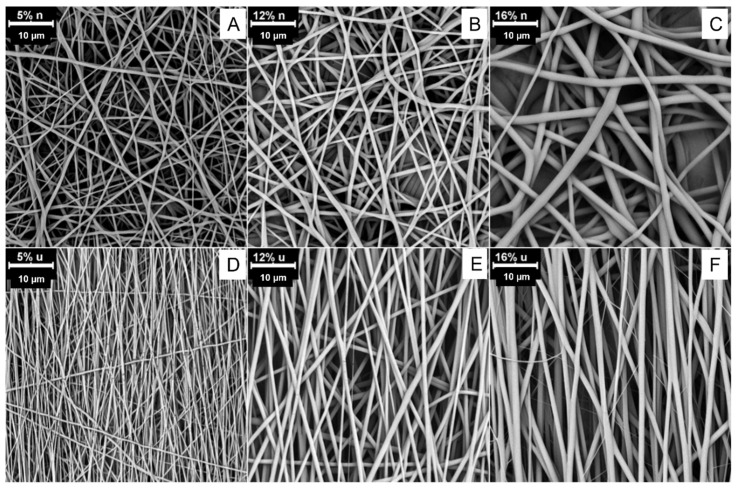
The morphology of obtained fibrous mats with a random arrangement of the fibers: (**A**) rPU5, (**B**) rPU12, and (**C**) rPU16 and aligned fibers (**D**) aPU5, (**E**) aPU12 and (**F**) aPU16.

SEM images showing the morphology of obtained electrospun fibers have been shown in Figure 2. Preliminary analysis of the acquired images indicates that the alignment of the fibers was obtained through the use of a collecting drum with high rotation speed. In addition, both nonwovens with undirected (random) (r) and aligned structures (a) show an increase in the fiber diameters with increasing concentration of the polyurethane within the solution (Table 1). In the case of a mat with aligned fibers, much smaller diameters and greater fiber uniformity are observed for each concentration.

It has been previously shown that for optimized parameters of electrospinning, increased polymer concentration causes an increase in the diameters of the fibers [28]. This is mainly attributed to the impact of the concentration on the viscosity and surface tension of the electrospinning solution [29].

**Figure 3:**
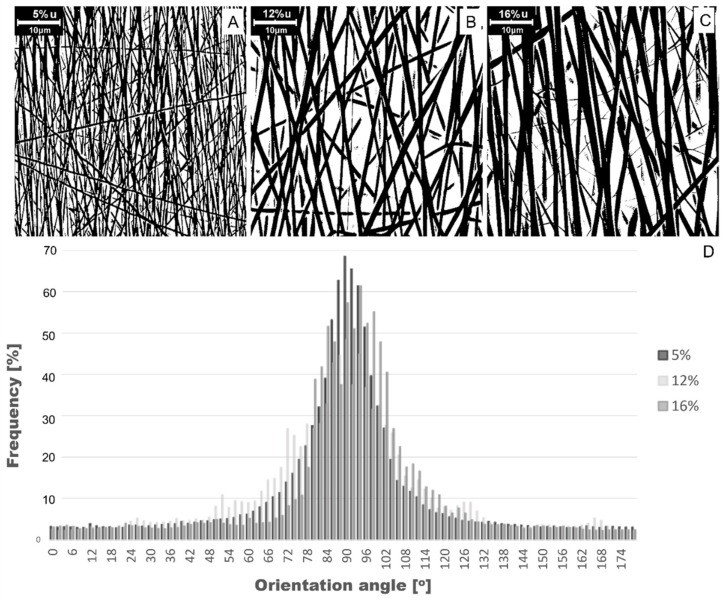
Morphology of the aligned fibrous structures obtained after binarization for SEM of (**A**) aPU5, (**B**) aPU12, and (**C**) aPU16; (**D**) histogram of average arrangement of aligned fibers.

Selected SEM images after binarization were employed to assess the degree of alignment and are presented in Figure 3A–C. Analysis of the images, which were taken in a manner intended to show the direction of the electrospinning, confirmed the presence of the direction-characterizing alignment. Each type of fabricated fiber has a dominant direction and a certain range over which alignment occurs. A histogram showing the distribution of the fibers is shown in Figure 3D. Table 2 shows the parameters describing the arrangement of the fibers in each type of mat. The angular range of the fiber’s alignment is between 60–120°, which means that fibers are arranged in a parallel direction with 30° deflection to this direction, which indicates a high alignment of the structure [30,31]. This makes it possible to assess the effect of the fiber alignment on the shape memory effect.

**Figure 4:**
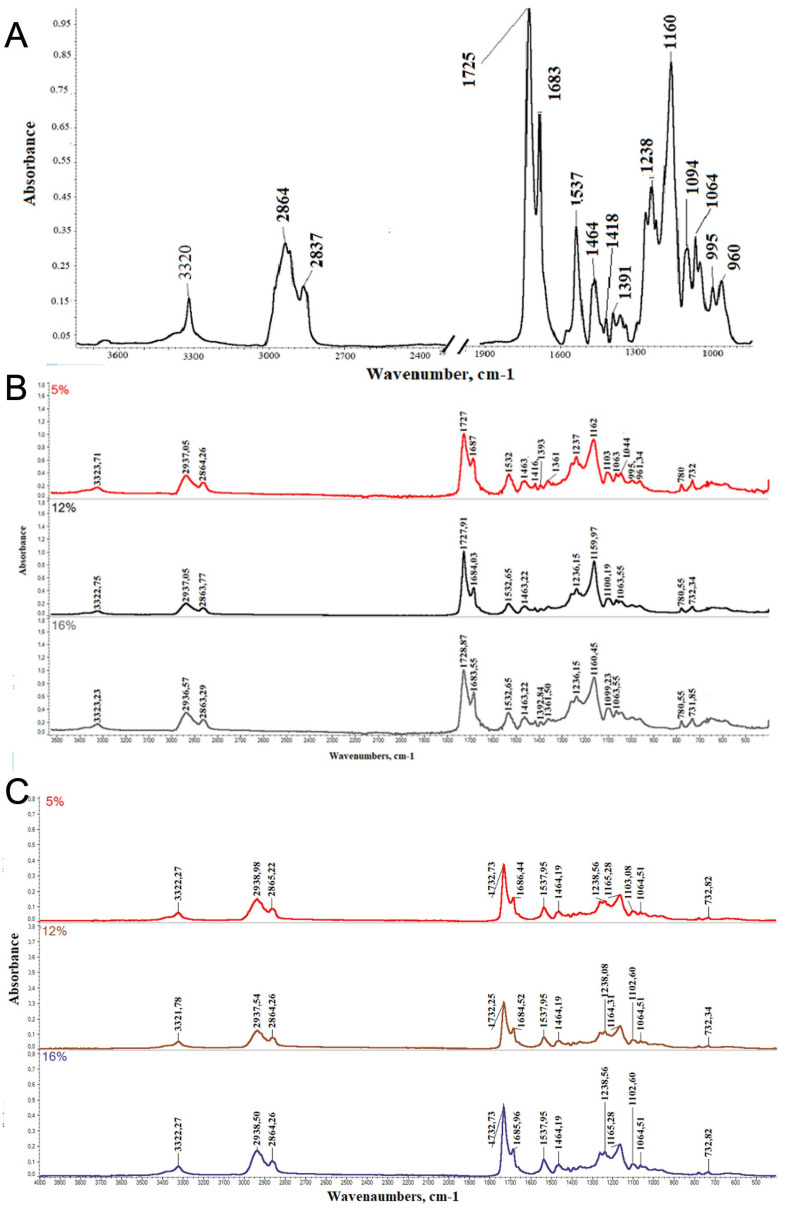
FTIR spectra of (**A**) pristine PU based on a mixture of PCL diols, (**B**) aligned mats PU and (**C**) random PU mats.

The chemical structure of the thermoplastic polyurethane composed of a mixture of PCL diols and aliphatic diisocyanate was analyzed by FTIR, and the spectra of obtained PU and electrospun mats are presented in Figure 4. FTIR analysis confirmed the presence of the urethane bonds characteristic for PU at 3320 cm^−1^, and 1725 cm^−1^ wavenumbers assigned to NH and C=O groups, respectively. Additionally, a peak at 1537 cm^−1^ characteristic for the second amide bond of urethane was observed. Moreover, the position of the peak associated with C=O groups and the additional arm at 1683 cm^−1^ confirmed that some number of carbonyl groups are associated with hydrogen bonds [32]. The hydrogen bonds may be formed within hard segments or between hard and soft segments of PU macromolecules. The presence of hydrogen bonds indicates the existence of micro-phase separation within the polymer segments, which may influence the mechanical and physical properties of PU [33]. These types of physical bonds, especially within the hard segments, were recognized and described in the literature as physical bonds that enhance mechanical and physical properties. Moreover, they are additional setpoints that improve shape memory properties [2,32]. Peaks characteristic for PCL segments were observed in the FTIR spectrum at the range of 1200 cm^−1^ to 1094 cm^−1^ and are associated with C-O-C of PCL diol, and bands at 2864–2837 cm^−1^ are characteristic for CH_2_.

FTIR spectra of electrospun mats presented in Figure 4B,C showed that the NH-associated band and carbonyl band are shifted towards higher wavenumbers, which indicates dissociation of some hydrogen bonds after electrospinning compared to pristine polyurethane.

**Figure 5:**
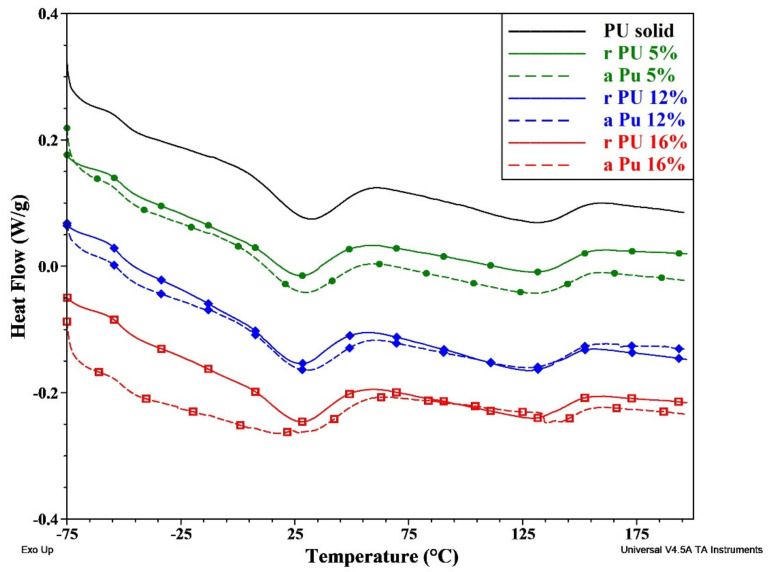
DSC 2nd heating curves for PU mats.

In the case of thermally induced and semicrystalline shape memory polymers, the transition temperature (T_trans_) from temporary to original/permanent shape is correlated with the melting temperature (Tm_s_) of the crystalline phase of switching PCL segments. The effect of electrospinning parameters on polymer structure, specifically on polymer crystallinity and the Tm_s_ of the PCL crystalline phase, was analyzed based on the second heating cycle thermograms (Figure 5). The DSC analysis results are summarized in Table 3. The DSC thermograms showed glass transition with T_g_ of −49 °C for pristine PU before processing and in the range of −50.7÷−52. °C for electrospun mats (Table 3). T_g_ for electrospun mats slightly decreased in comparison to pristine PU, which suggests that micro-phase separation between soft and hard segments of the PU macromolecules was higher in electrospun fibers made of PU than in bulk PU [34]. Additional reasons for the slight decrease in T_g_ for various electrospun polymers could be associated with increasing segmental mobility due to lower chain entanglements [21].

DSC thermograms displayed the presence of two endothermic transitions for all samples that are correlated with the melting of the crystalline phase of soft and hard segments of PU macromolecules. The crystalline phase assigned to the PCL soft/switching segments exhibited a melting temperature (Tm_s_) in the range of 27–30 °C and slightly decreased after electrospinning when compared to pristine polyurethane (Table 3). The values of Tm_s_ for random and aligned fibers are very similar, although insignificantly higher Tm_s_ was observed for an aligned mat. Similarly, there was no clear (increasing or decreasing) trend for Tm_s_ of electrospun mats correlated with increasing concentration of PU. The crystallinity of the PCL soft segments was evaluated based on measured values of ΔH J/g, and the highest value of 8.6% was found for solid PU, and the slight decrease was observed for the electrospun mats with the lowest value of 6.5% for rPU5. The obtained results are in good agreement with previously reported data that showed decreasing crystallinity and Tm value of electrospun mats in comparison to solid polymers [19,20].

For electrospun fibers, two opposite factors influence the crystallization process. While fast evaporation of solvent may limit molecular mobility and thus the ability of macromolecules for crystallization, the fiber alignment may enhance crystallization [35,36]. The lower Tm value of electrospun mats in comparison to pristine PU film indicates the formation of smaller crystallites with more defects, probably due to faster evaporation of the solvent and restricted chain mobility [19,20,21]. However, for aPU mats, where there were more macromolecules oriented in one direction (Table 2), crystallization was easier and more crystals with the perfect structure were formed when compared to random mats. Therefore, slightly higher Tm_s_ and crystallinity (χ%) were observed for aPU mats. The shape of the melting peak of soft/switching segments is quite broad and comparable for all samples, which indicates the heterogeneous morphology of the soft segments’ crystallites (Figure 5). It is a consequence of mixing PCL diols of various molecular weights that forms various lengths of PCL segments with different mobility and crystallization rates. Very broad endothermic peaks with Tm_h_ value in the range of 126–135 °C are correlated with melting of crystalline phase or dissociation of some short order domains within hard segments of PU. Very broad peak indicates on high imperfection of crystallites and some extent of phase mixing within PU structure [37]. The values of enthalpy of fusion are slightly higher for hard segments in comparison to soft segments (Table 3), which may indicate a higher interaction within the hard segments of PU. It is justified as hard segments are shorter with higher concentration of reactive functional groups. The presence of crystalline phase with melting temperature above T_trans_ may enhance shape memory effect. Summarizing DSC results it could be concluded that electrospinning process using solution concentration in the range of 5–16 (wt.%) and voltage in the range of 11–20 kV did not change significantly Tm or crystallinity of used polyurethane.

The contact angle measurements (Table 4.) revealed the hydrophobic nature of the fibers regarding their arrangement. The water contact angle (WCA) evaluated for random fibrous mates was in the range of 114–133° for random fibers and 106–126° for aligned. It has been observed that the arrangement of the fibers has a strong influence on the hydrophilicity of the fibers [38]. However, for hydrophobic materials such as polyurethanes used in this study, although a decrease within the contact angle is significant, it does not cause a change of the character of the surface of the fibrous mat from hydrophobic to hydrophilic.

**Figure 6:**
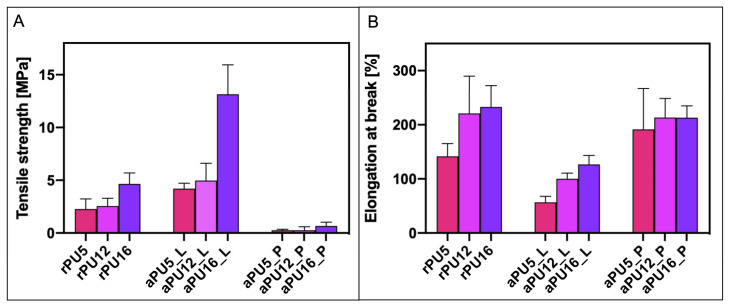
Comparison of (**A**) tensile strength and (**B**) strain at break values for investigated mats.

The mechanical properties evaluation for random and aligned fibers has been shown in Figure 6. Mechanical strength for random fibers was 2.27 ± 0.96 MPa for rPU5, 2.55 ± 0.66 MPa for rPU12 and 4.66 ± 1.03 MPa for rPU16, while the tensile strain was 141.68 ± 23.54% for rPU5, 220.81 ± 69.00% for rPU12 and 233.00 ± 39.33% for rPU16. The aligned orientated fibers were tested in two directions, parallel and longitudinal, to the direction of the fibers. The tensile strength in longitudinal direction was 4.21 ± 0.52 MPa for aPU5, 4.98 ± 1.64 MPa for aPU12 and 13.15 ± 3.01 MPa for aPU16, while tensile strain was 56.87 ± 11.01% for aPU5, 100.09 ± 10.65 MPa for aPU12 and 126.61 ± 16.73 MPa for aPU16. Moreover, tensile strength in parallel direction was measured at 0.26 ± 0.09 MPa for aPU5, 0.27 ± 0.29 MPa for aPU12, and 0.66 ± 0.36 MPa for aPU16, while strain was 191.59 ± 75.31% for aPU5, 213.38 ± 35.16% for aPU12 and 213.07 ± 21.81% for aPU16. The tensile stress increased with the diameter of the fibers. Moreover, as was expected, obtained mechanical stress values for the aligned fibers tested in a longitudinal direction were higher compared to random fibers made from the same solutions. It was previously reported that the diameter and the alignment of the fibers increase the mechanical strength of the polymeric fibrous structures [24]. The decrease in the mechanical properties when performing the tensile tests parallel to the direction of the fibers might be attributed to the delamination of the fibrous structure.

**Figure 7:**
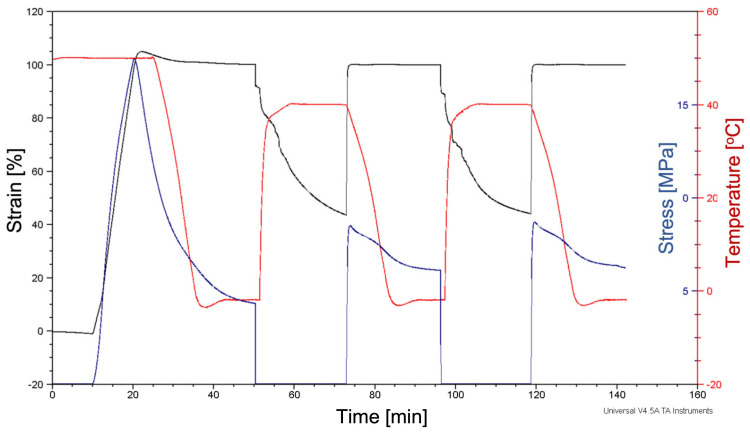
The flow of the programming process registered on DMA for rPU16 mat.

The cyclic thermomechanical process presented in Figure 7 was applied to investigate the shape-memory effect of the electrospun mats. The values of R_r_ and Rf coefficients calculated based on DMA analysis are presented in Table 5 and Table 6, respectively. After the first cycle of deformation and recovery in the air environment, R_r_ values for mats with a random arrangement of fibers were in the range from 57% for rPU16 to 60% for rPU5, respectively. Contrary, for aligned mats, the lowest value of the R_r_ coefficient, measured after the first shape recovery cycle in the air, was found for aPU5 mats and slightly increased for aPU12 and aPU16, respectively. Additionally, no significant change was observed after the second cycle of the shape recovery process. However, after the 3rd cycle of shape programming and recovery process performed in the water environment at 40 °C significant increase in R_r_ value was observed for both random and aligned mats for all solution concentrations (Table 5). These findings indicate that developed mats may be considered for medical applications where the water environment and temperature transition to the original shape around 40 °C are conditions similar to those tested within this manuscript.

The obtained results did not indicate a clear correlation between fiber diameters and shape memory properties. Similarly, various conclusions are reported in the literature regarding the effect of electrospun fiber diameter on shape memory properties. For example, Sauter et al. [17] reported that smaller fiber diameters in the nanoscale below 100 nm enhance shape memory properties due to a higher percent of molecular orientation along with fiber elongation direction and higher stress generated in fibers with lower diameter. However, the same authors declared that this mechanism is clearly seen only for fibers on a small nanometer scale. Taking into account that all fibers in our mats were in submicron and micron scale, the effect of fiber diameter on shape recovery could not be clearly seen. Similar observations were presented by Banikazemi et al. [39]. An opposite correlation between fiber diameter and shape, recovery was reported by Xi et al. for acrylate-based electrospun mats [18].

In order to explain an increase in R_r_ value after the third cycle, various factors need to be concerned. First of all, it is often observed that R_r_ increase with an increasing number of thermomechanical cycles due to a higher amount of stored energy within the polymer matrix. Additionally, the plasticizing effect of water should be considered. In the presence of water, macromolecules are more labile, and therefore even with the same level of internal energy stored in setpoints of hard segments, relaxation and recovery become faster. Therefore, at the same time and temperature, a much higher shape recovery was recorded. The shape recovery process is driven by the entropic energy or internal stress that was stored in frozen deformed chains. After heating, macromolecules gain more mobility, which releases the entropic energy and chains back into its highest entropic energy associated with the permanent shape [40]. In the case of aPU mats, more particular fibers were subjected to elongation applied along the same axis, which resulted in higher internal stress accumulated in the macromolecules. Therefore, the shape recovery process is faster and more efficient. In the case of random fibers, when the sample was deformed, some fibers that were transversely oriented to the elongation direction were not deformed in the same manner as longitudinal fibers, and thus less internal stress was accumulated in the sample. This resulted in a lower shape recovery rate.

Table 6 shows the shape fixity of rPU and aPU electrospun mats. The shape fixity of electrospun mats is higher than 90% in the three cycles for aPU. Some decrease in R_f_ was observed for rPU samples after the third cycle. It could be connected with some chain entanglement and restricted crystallization with a higher number of thermos-programming cycles. However, taking into account that all samples have similar crystallinity, a similar R_f_ was expected. Overall, these results demonstrate that the electrospun mats, especially with aligned fibers, have good shape-fixed performance.

## 4. Conclusions

Electrospun mats with versatile diameters of the fibers with the random and aligned arrangement of the fibers within the structures were fabricated from shape memory PU comprising a mixture of PCL polyols as soft segments. The diameters were controlled by changes in a solution concentration of PU within the range of 5–16 wt.%. The thermal analysis DSC showed that the crystalline structure of soft and hard segments and melting temperature were not significantly affected by the electrospinning process. These results indicate that a carefully optimized electrospinning process does not change the previously designed structure of the polyurethane. Fiber diameter in the range of 410 to 1760 nm and from 360 to 1200 nm for random and aligned mats, respectively, did not influence crystallinity or shape memory evaluated in the air conditions. The more substantial effect of the fiber alignment on shape recovery was observed for samples tested in water. Shape recovery for aligned fibers performed in the water was in the range of 84–96%, while for random mats tested in the same conditions were 10 to 20% lower depending on fiber diameter. These findings show that fiber alignment and thermo-mechanical programming may enhance the shape memory performance of electrospun mats.

## Figures and Tables

**Table 1:** The average diameter of obtained fibers.

Concentration [%]	Average Diameter for Random Fibers [nm]	Average Diameter for Aligned Fibers [nm]
5	410 ± 90	360 ± 70
12	1160 ± 260	890 ± 130
16	1760 ± 540	1200 ± 210

**Table 2:** The degree of the fibers’ alignment within the structures.

Fibers	Dominant Direction [°]	Contribution in Range of 60–120° [%]
aPU5	89.21 ± 8.14	78 ± 2
aPU12	87.54 ± 12.19	74 ± 1
aPU16	86.72 ± 9.10	80 ± 2

**Table 3:** Summary of the characteristic temperatures DSC results of tested materials.

Temperature		Tg [°C]	Tm_s_ [°C]	Tm_h_ [°C]	ΔHm_s_ [J/g]	Χ, %	ΔHm_h_ [J/g]
Pristine polyurethane		−49.0	30.8	130.8	11.7	8,6	11.1
Random	rPU5	−50.7	27.5	130.9	8.9	6.5	11.1
	rPU12	−52.7	26.8	126.9	10.8	7.9	12.3
	rPU16	−51.4	27.4	128.9	10.9	8.0	10.5
Aligned	aPU5	−52.4	28.4	127.1	10.9	8.0	11.0
	aPU12	−51.8	28.8	127.1	9.7	7.1	12.1
	aPU16	−51.5	27.4	135.4	10.6	7.8	11.9

**Table 4:** The hydrophilicity of the tested samples.

Sample	Contact Angle [°]	Shape of the Water Droplet
rPU5	114 ± 4	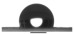
rPU12	132 ± 5	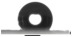
rPU16	133 ± 3	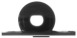
aPU5	106 ± 7	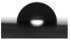
aPU12	126 ± 2	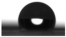
aPU15	122 ± 4	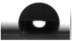

**Table 5:** Values of recovery rate (R_r_).

Sample	Arrangement of Fibers	Cycle I	Cycle II	Cycle III (in Water)
rPU5	Random	60.0 ± 5.9	59.0 ± 6.46	73.8 ± 4.36
aPU5	Longitudinal direction *	58.5 ± 2.12	57.4 ± 1.62	84.8 ± 3.21
rPU12	Random	58.7 ± 0.91	58.5 ± 0.99	75.6 ± 3.10
aPU12	Longitudinal direction *	60.1 ± 1.48	59.4 ± 1.36	86.9 ± 0.64
rPU16	Random	57.3 ± 2.59	56.4 ± 3.21	74.3 ± 3.66
aPU16	Longitudinal direction *	60.0 ± 3.59	58.9 ± 5.96	95.67 ± 1.36

*—targeted programming in the longitudinal direction to the fiber alignment.

**Table 6:** Values of shape fixity.

Sample	Arrangement of Fibers	Cycle I	Cycle II	Cycle III
rPU5	Random	93.10 ± 2.05	94.15 ± 0.39	91.0
aPU5	Longitudinal direction *	95.18 ± 0.99	94.76 ± 0.61	93.52 ± 1.69
rPU12	Random	95.49 ± 0.35	93.66 ± 2.04	87.26 ± 3.64
aPU12	Longitudinal direction *	95.12 ± 0.36	94.15 ± 0.40	95.72 ± 1.24
rPU16	Random	92.69 ± 2.03	93.30 ± 1.91	87.0 ± 3.41
aPU16	Longitudinal direction *	93.75 ± 1.10	92.97 ± 3.24	96.95 ± 1.57

* targeted programming in the longitudinal direction to the fiber alignment.

## Data Availability

Not applicable.

## References

[1] Delaey J., Dubruel P., Van Vlierberghe S. (2020). Shape-Memory Polymers for Biomedical Applications. Adv. Funct. Mater..

[2] Bil M., Kijeńska-Gawrońska E., Głodkowska-Mrówka E., Manda-Handzlik A., Mrówka P. (2020). Design and in Vitro Evaluation of Electrospun Shape Memory Polyurethanes for Self-Fitting Tissue Engineering Grafts and Drug Delivery Systems. Mater. Sci. Eng. C.

[3] Wong Y.S., Stachurski Z.H., Venkatraman S.S. (2011). Modeling Shape Memory Effect in Uncrosslinked Amorphous Biodegradable Polymer. Polymer.

[4] Salaris V., Leonés A., Lopez D., Kenny J.M., Peponi L. (2022). Shape-Memory Materials via Electrospinning: A Review. Polymers.

[5] Gupta A., Maharjan A., Kim B.S. (2019). Shape Memory Polyurethane and Its Composites for Various Applications. Appl. Sci..

[6] Paik I.H., Goo N.S., Jung Y.C., Cho J.W. (2006). Development and Application of Conducting Shape Memory Polyurethane Actuators. Smart Mater. Struct..

[7] Zhang L., Huang Y., Dong H., Xu R., Jiang S. (2021). Flame-Retardant Shape Memory Polyurethane/MXene Paper and the Application for Early Fire Alarm Sensor. Compos. Part B Eng..

[8] Sáenz-Pérez M., Bashir T., Laza J.M., García-Barrasa J., Vilas J.L., Skrifvars M., León L.M. (2019). Novel Shape-Memory Polyurethane Fibers for Textile Applications. Text. Res. J..

[9] Gabriel L.P., Rodrigues A.A., Macedo M., Jardini A.L., Maciel Filho R. (2017). Electrospun Polyurethane Membranes for Tissue Engineering Applications. Mater. Sci. Eng. C.

[10] Bil M., Hipś I., Mrówka P., Święszkowski W. (2020). Studies on Enzymatic Degradation of Multifunctional Composite Consisting of Chitosan Microspheres and Shape Memory Polyurethane Matrix. Polym. Degrad. Stab..

[11] Puszka A. Poliuretany—Surowce, Właściwości Oraz Modyfikacje. https://docplayer.pl/6805689-Poliuretany-surowce-wlasciwosci-oraz-modyfikacje.html.

[12] Gu L., Cui B., Wu Q.-Y., Yu H. (2016). Bio-Based Polyurethanes with Shape Memory Behavior at Body Temperature: Effect of Different Chain Extenders. RSC Adv..

[13] Garg H., Mohanty J., Gupta P., Das A., Tripathi B.P., Kumar B. (2020). Polyethylenimine-Based Shape Memory Polyurethane with Low Transition Temperature and Excellent Memory Performance. Macromol. Mater. Eng..

[14] Mao H.-I., Chen C.-W., Guo L.-Y., Rwei S.-P. (2022). Tunable Shape Memory Property Polyurethane with High Glass Transition Temperature Composed of Polycarbonate Diols. J. Appl. Polym. Sci..

[15] Zhu Y., Hu J., Choi K.-F., Yeung K.-W., Meng Q., Chen S. (2008). Crystallization and Melting Behavior of the Crystalline Soft Segment in a Shape-Memory Polyurethane Ionomer. J. Appl. Polym. Sci..

[16] Zhuo H., Hu J., Chen S. (2008). Electrospun Polyurethane Nanofibres Having Shape Memory Effect. Mater. Lett..

[17] Sauter T., Kratz K., Heuchel M., Lendlein A. (2021). Fiber Diameter as Design Parameter for Tailoring the Macroscopic Shape-Memory Performance of Electrospun Meshes. Mater. Des..

[18] Xi J., Shahab S., Mirzaeifar R. (2022). Qualifying the Contribution of Fiber Diameter on the Acrylate-Based Electrospun Shape Memory Polymer Nano/Microfiber Properties. RSC Adv..

[19] Mujica-Garcia A., Navarro-Baena I., Kenny J., Peponi L. (2014). Influence of the Processing Parameters on the Electrospinning of Biopolymeric Fibers. J. Renew. Mater..

[20] Kolbuk D., Guimond-Lischer S., Sajkiewicz P., Maniura-Weber K., Fortunato G. (2015). The Effect of Selected Electrospinning Parameters on Molecular Structure of Polycaprolactone Nanofibers. Int. J. Polym. Mater. Polym. Biomater..

[21] Ero-Phillips O., Jenkins M., Stamboulis A. (2012). Tailoring Crystallinity of Electrospun Plla Fibres by Control of Electrospinning Parameters. Polymers.

[22] Kijeńska E., Prabhakaran M.P., Swieszkowski W., Kurzydlowski K.J., Ramakrishna S. (2012). Electrospun Bio-Composite P(LLA-CL)/Collagen I/Collagen III Scaffolds for Nerve Tissue Engineering. J. Biomed. Mater. Res. Part B Appl. Biomater..

[23] Kijeńska-Gawrońska E., Swieszkowski W. (2017). General Requirements of Electrospun Materials for Tissue Engineering. Electrospun Materials for Tissue Engineering and Biomedical Applications: Research, Design and Commercialization.

[24] Kijeńska-Gawrońska E., Bolek T., Bil M., Swieszkowski W. (2019). Alignment and Bioactive Molecule Enrichment of Bio-Composite Scaffolds towards Peripheral Nerve Tissue Engineering. J. Mater. Chem. B.

[25] Katta P., Alessandro M., Ramsier R.D., Chase G.G. (2004). Continuous Electrospinning of Aligned Polymer Nanofibers onto a Wire Drum Collector. Nano Lett..

[26] Borisova I., Stoilova O., Manolova N., Rashkov I. (2021). Effect of Coating on the Mechanical Properties of Electrospun Poly(3-Hydroxybutyrate) Materials with Targeted Fibers Alignment. J. Polym. Res..

[27] Zhu G., Xu Q., Qin R., Yan H., Liang G. (2005). Effect of γ-Radiation on Crystallization of Polycaprolactone. Radiat. Phys. Chem..

[28] Doshi J., Reneker D.H. (1995). Electrospinning Process and Applications of Electrospun Fibers. J. Electrost..

[29] Deitzel J.M., Kleinmeyer J., Harris D., Beck Tan N.C. (2001). The Effect of Processing Variables on the Morphology of Electrospun Nanofibers and Textiles. Polymer.

[30] Theron A., Zussman E., Yarin A.L. (2001). Electrostatic Field-Assisted Alignment of Electrospun Nanofibres. Nanotechnology.

[31] Kim K.W., Lee K.H., Khil M.S., Ho Y.S., Kim H.Y. (2004). The Effect of Molecular Weight and the Linear Velocity of Drum Surface on the Properties of Electrospun Poly(Ethylene Terephthalate) Nonwovens. Fibers Polym..

[32] Bil M., Mrówka P., Kołbuk D., Święszkowski W. (2021). Multifunctional Composite Combining Chitosan Microspheres for Drug Delivery Embedded in Shape Memory Polyester-Urethane Matrix. Compos. Sci. Technol..

[33] Bil M., Ryszkowska J., Woźniak P., Kurzydłowski K.J., Lewandowska-Szumieł M. (2010). Optimization of the Structure of Polyurethanes for Bone Tissue Engineering Applications. Acta Biomater..

[34] Son T.W., Lee D.W., Lim S.K. (1999). Thermal and Phase Behavior of Polyurethane Based on Chain Extender, 2,2-Bis-[4-(2-Hydroxyethoxy)Phenyl]Propane. Polym. J..

[35] Echeverría C., Limón I., Muñoz-Bonilla A., Fernández-García M., López D. (2021). Development of Highly Crystalline Polylactic Acid with β-Crystalline Phase from the Induced Alignment of Electrospun Fibers. Polymers.

[36] Zhou H., Green T.B., Joo Y.L. (2006). The Thermal Effects on Electrospinning of Polylactic Acid Melts. Polymer.

[37] Szycher M. (2012). Structure–Property Relations in Polyurethanes. Szycher’s Handbook of Polyurethanes.

[38] Areias A.C., Ribeiro C., Sencadas V., Garcia-Giralt N., Diez-Perez A., Gómez Ribelles J.L., Lanceros-Méndez S. (2012). Influence of Crystallinity and Fiber Orientation on Hydrophobicity and Biological Response of Poly(l-Lactide) Electrospun Mats. Soft Matter.

[39] Banikazemi S., Rezaei M., Rezaei P., Babaie A., Eyvazzadeh-Kalajahi A. (2020). Preparation of Electrospun Shape Memory Polyurethane Fibers in Optimized Electrospinning Conditions via Response Surface Methodology. Polym. Adv. Technol..

[40] Zhao Q., Qi H.J., Xie T. (2015). Recent Progress in Shape Memory Polymer: New Behavior, Enabling Materials, and Mechanistic Understanding. Prog. Polym. Sci..

